# Advances in Chiral Pincer Complexes: Insights and Applications in Catalytic Asymmetric Reactions †

**DOI:** 10.3390/ijms251910344

**Published:** 2024-09-26

**Authors:** Sanaa Musa, Yuval Peretz, Gil Dinnar

**Affiliations:** 1 Natural Compounds and Organic Synthesis Laboratory, Migal-Galilee Research Institute, Kiryat Shmona 11016, Israel; 2 Department of Biotechnology, Tel-Hai Academic College, Kiryat Shmona 11016, Israel

**Keywords:** chiral-type ligands, chiral pincer complexes, asymmetric catalysis, enantioselectivity, enantioenriched compounds

## Abstract

Chiral pincer complexes, characterized by their rigid tridentate coordination framework, have emerged as powerful catalysts in asymmetric synthesis. This review provides a comprehensive overview of recent advancements in the development of chiral pincer-type ligands and their corresponding transition metal complexes. We highlight the latest progress in their application across a range of catalytic asymmetric reactions, including the (transfer) hydrogenation of polar and non-polar bonds, hydrophosphination, alkynylation, Friedel-Crafts reactions, enantioselective reductive cyclization of alkynyl-tethered cyclohexadienones, enantioselective hydrosilylation, as well as Aza–Morita–Baylis–Hillman reactions. The structural rigidity and tunability of chiral pincer complexes enable precise control over stereoselectivity, resulting in high enantioselectivity and efficiency in complex molecular transformations. As the field advances, innovations in ligand design and the exploration of new metal centers are expected to expand the scope and utility of these catalysts, bearing significant implications for the synthesis of enantioenriched compounds in pharmaceuticals, materials science, and beyond.

## 1. Introduction

Organometallic pincer complexes, characterized by their tridentate ligands, are known for creating a rigid steric and electronic coordination environment around the metal center. This structural rigidity enables the formation of species with versatile reactivities, which can be fine-tuned through modifications to the ligand framework [1]. These properties have led to their successful application across various branches of catalysis and coordination chemistry, expanding into fields such as organic materials, bioinorganic chemistry, and supramolecular chemistry [2,3,4,5]. More importantly, the ability to fine-tune the structure environment around the metal center makes them highly attractive for developing chiral pincer complexes and applying them to a diverse array of asymmetric organic transformations [6,7].

As the demand for more efficient and selective catalytic processes continues to grow, the role of chiral pincer complexes has become increasingly prominent. These complexes not only offer the possibility of achieving high levels of enantioselectivity but also provide the robustness required for challenging catalytic conditions. Moreover, their versatility allows for the exploration of a wide range of catalytic reactions, from well-established processes to novel transformations that require precise control over stereochemistry. Over the past years, various asymmetric transformations have been developed using chiral pincer complexes, including but not limited to asymmetric (transfer) hydrogenation [8,9], alkynylation [10], borylation of α,β-unsaturated carbonyl compounds [11], allylation and propargylation reactions of aldehydes [12,13], aldol condensation [14,15], hydrophosphination of α,β-unsaturated carbonyl compounds with R_2_PH [16], condensation of sulfonimines and isocyanoacetate [17], allylation of ketimines [18], Diels–Alder [19], as well as the Friedel−Crafts reaction of indoles [20].

In this review, we aim to provide a comprehensive overview of the latest advancements in the design and application of chiral pincer-type ligands and their transition metal complexes. We will explore how these systems have been engineered to enhance enantioselectivity and efficiency in various catalytic reactions, underscoring their evolving role in asymmetric catalysis. Through a detailed examination of the recent literature, this review will highlight the potential of chiral pincer ligands to drive future innovations in asymmetric synthetic chemistry, contributing to the ongoing development of progressively sophisticated and selective catalytic processes.

## 2. Enantioselective Hydrogenation of C=O Bonds 

### 2.1. Asymmetric Hydrogenation 

**Figure 1:**
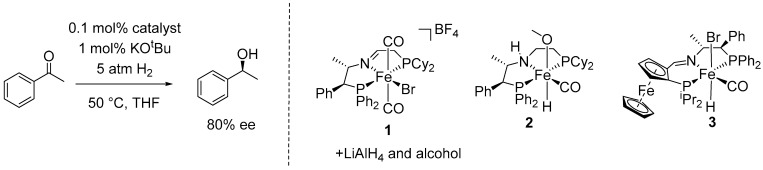
Selected chiral pincer complexes developed by Morris for the asymmetric hydrogenation of acetophenone.

The asymmetric hydrogenation (AH) of unsaturated double bonds is a fundamental process for converting prochiral compounds into chiral molecules [21]. This groundbreaking process was introduced by Knowles et al. in 1986, who paved the way to produce enantioenriched chiral compounds. In the 1990s, Noyori and co-workers significantly advanced the field by establishing foundational methodologies for asymmetric hydrogenation, which have since become essential in asymmetric organic synthesis [22,23,24]. Following these advancements, numerous catalytic systems incorporating chiral ligands have been developed [9,21]. Interestingly, various research groups have independently developed a set of chiral tridentate pincer ligands for the asymmetric hydrogenation of ketones, which afforded excellent results in terms of enantioselectivity [25,26,27,28]. Notably, the group of Morris was the first to publish a chiral active iron (II) AH catalyst system derived from precursor complexes of the type [Fe(P-N-P’)(CO)_2_(Br)]BF_4_ (**1**) with the unsymmetrical pincer ligand PNP’. When treated with LiAlH_4_, alcohol, and a base, precatalyst **1** yielded an active and enantioselective catalyst for the AH of prochiral ketones to alcohols (up to 89% enantiomeric excess (ee)) in tetrahydrofuran (THF) under mild conditions (22–50 °C, 5–20 atm H_2_) [29,30]. Subsequently, the same group developed two additional improved chiral iron (II) PNHP’(CO)(H)(OMe) (**2**) and PNP’ pincer ligands with a scaffold consisting of a planar chiral ferrocene and a central chiral aliphatic unit (**3**), achieving enantioselectivities for the AH of aromatic prochiral ketones of up to 96% ee (Figure 1) [31,32]. 

**Figure 2:**
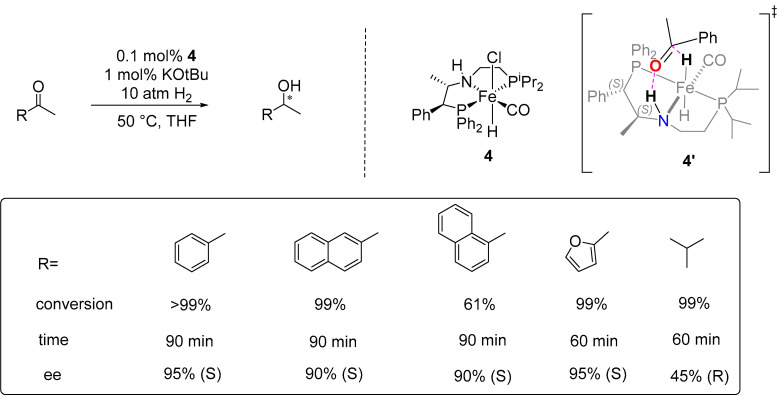
Asymmetric hydrogenation catalyzed by catalyst **4**, which was developed by Morris in 2017. * indicates chiral center, and ^‡^ represnts transition state.

Additionally, they developed an improved iron (II) (P-NH-P’) pincer catalyst (**4**), which showed great promise for the enantioselective AH of ketones. Catalyst **4** enabled the quantitative AH of a variety of prochiral aryl ketones to enantiomerically enriched (*S*)-alcohols in up to 96% ee value under mild conditions (THF, 0.1 mol% of **4**, 1 mol% of KO^t^Bu, 5–10 atm H_2_, 50 °C) (Figure 2) [33]. Density functional theory (DFT) calculations provided the transition state (**4′**), producing (*S*)-1-phenylethanol by the attack of a hydride in trans-FeH_2_(CO)(P-NH-P’) on acetophenone, which was found to be 1.6 kcal/mol lower in energy than that producing the (*R*)-alcohol [31]. In the suggested structure, the phenyl group of the ketone is positioned above the isopropyl groups, while the smaller methyl group is oriented against a phenyl of the PPh_2_. This phenyl group is located in the position by the adjacent asymmetric array of phenyl groups, which also locked the five-membered -PCHPhCHPhNHFe- ring by taking the favorable equatorial positions (Figure 2). 

**Figure 3:**
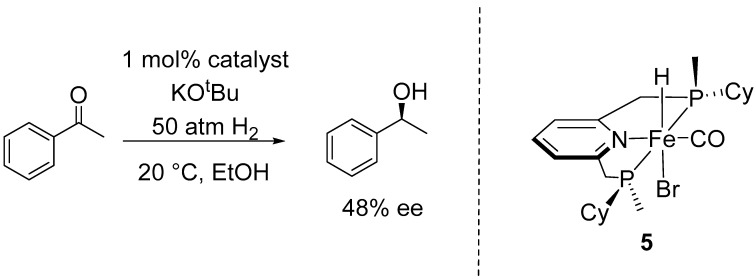
Asymmetric hydrogenation of acetophenone developed by Mezzetti.

In 2018, Mezzetti et al. developed a chiral iron (II) PNP pincer complex bearing P-stereogenic centers (**5**), analogous to the well-known Milstein’s catalyst [34]. In the presence of a base, such as potassium tert-butoxide (^t^BuOK), complex **5** catalyzed the asymmetric hydrogenation of acetophenone to (*S*)-1-phenylethanol with 48% ee (Figure 3). Their study included an analysis of the transition states involved in the enantiodetermining transfer of a hydride from complex **5** to the carbonyl group of acetophenone using DFT. The DFT calculations revealed that the outer-sphere monohydride mechanism, initially proposed by Milstein, accurately predicted the experimentally observed sense of induction (*S*) and enantioselectivity, whereas the dihydride and inner-sphere pathways would lead to the formation of the *R*-enantiomer [27].

**Figure 4:**
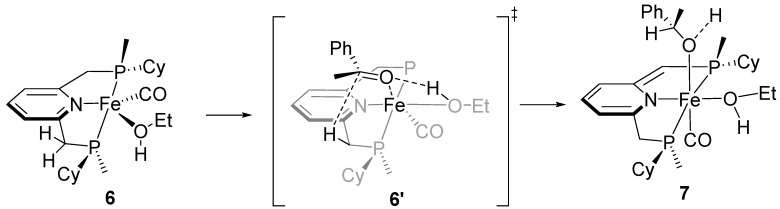
Proposed mechanism of the enantiodetermining step of AH.

The suggested outer-sphere mechanism involves the Fe (0) complex **6**, from which a benzylic H atom of the PNP ligand is transferred as a hydride to the carbonyl group of acetophenone (**6′**). A hydrogen bond involving the coordinated ethanol molecule directs the incoming substrate and activates its carbonyl group toward nucleophilic attack (**7**) [34,35], which is reminiscent of the well-established bifunctional mechanism (Figure 4) [36,37,38]. 

**Figure 5:**
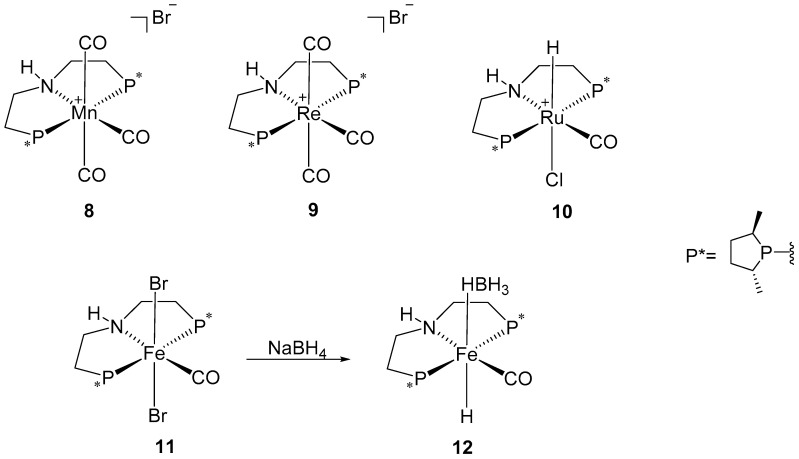
Chiral metal pincer complexes were developed by Beller’s research group, with P* presenting the chiral phosphine ligand.

Later on, Beller and co-workers reported the synthesis of different chiral metal pincer complexes using chiral bis(2-((2*R*,5*R*)-2,5-dimethylphospholanoethyl))amine as the ligand, and manganese (Mn), rhenium (Re), ruthenium (Ru), and iron (Fe) as metal centers (Figure 5) [39,40]. Their studies demonstrated that the non-noble Mn and Fe complexes (**8** and **11**, respectively) outperformed the related Re and Ru catalysts (**9** and **10**, respectively). Catalyst **8** achieved full conversion for both acetophenone and cyclohexyl methyl ketone, with an 84% ee for the latter substrate. Interestingly, catalyst **11** and its active complex, **12**, exhibited nearly the same selectivity for both substrates; however, complex **12** showed higher activity in reducing cyclohexyl methyl ketone with a 62% ee of the *R*-configuration. When comparing catalysts **8** and **12** with various aliphatic prochiral ketones, both showed high activity, but catalyst **8** exhibited a higher enantiomeric excess (up to 99% ee).

**Figure 6:**
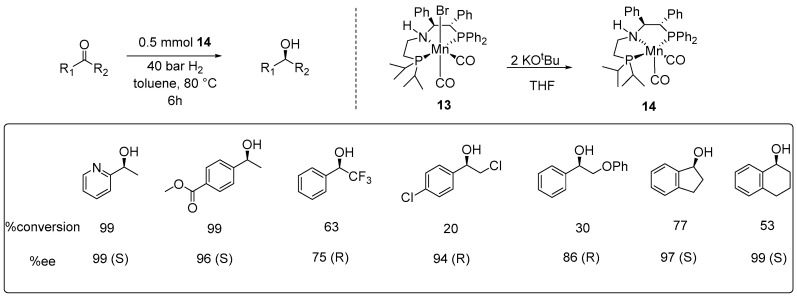
Asymmetric hydrogenation catalyzed by catalyst **14**, as developed by Morris in 2021.

Subsequently, Morris and colleagues developed an amidophosphine manganese catalyst Mn(CO)_2_(P-N-P’)(**14**) using a homochiral, unsymmetrical pincer ligand (*S*,*S*)-PPh_2_CHPhNHCH_2_CH_2_PiPr_2_ (P-NH-P’), analogous to the iron complex (**4**) previously reported by the same group (Figure 2) [33]. In their improved approach, they synthesized, characterized, and isolated a new species from Mn(Br)(CO)_2_PNP’ (**13**) through a rapid dehydrohalogenation reaction using KO^t^Bu in THF (Figure 6), which was proposed to be an intermediate involved in the catalytic cycle [41]. Catalyst **14** demonstrated exceptionally high enantioselectivity and good activity in base-free asymmetric ketone hydrogenation, with a tolerance for base-sensitive substrates. For example, 2,4′-dichloroacetophenone and 2-phenoxyacetophenone were hydrogenated in low conversion (20% and 30%, respectively) but with high ee. 

**Figure 7:**
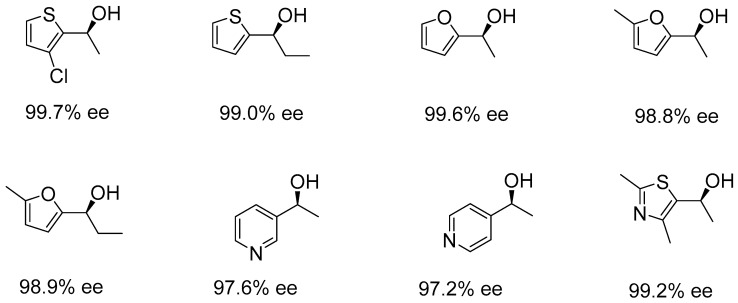
Selected heteroaromatic chiral alcohols catalyzed by cinchona alkaloid-based NNP pincer (conversion >99%), as developed by Zhang et al.

In 2022, Zhang et al. developed a series of cinchona alkaloid-based NNP pincer ligands, which were subsequently used with a ruthenium precursor in the asymmetric hydrogenation of ketones. The catalytic reactions were carried out using the in situ generation of the NNP-Ru pincer complex under 60 atm H_2_ in methanol at 30 °C. Several ketone substrates were reduced smoothly to afford the corresponding chiral alcohols with high enantioselectivities (up to 96%), including aryl alkyl and various heteroaromatic ketones containing nitrogen, oxygen, or sulfur (Figure 7) [42]. They demonstrated that while the catalytic activity of the ruthenium catalyst was lower than that of their previously developed iridium catalyst using the same ligand, the enantioselectivity was maintained and, in some cases, even surpassed that of the iridium catalyst in the hydrogenation of certain substrates [43].

**Figure 8:**
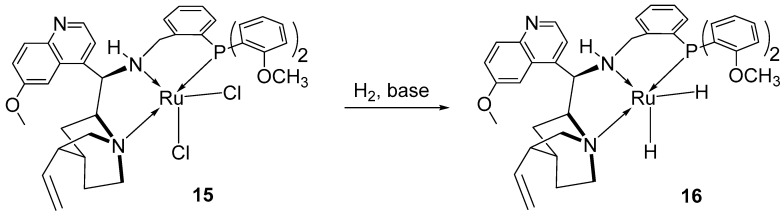
The NNP-ruthenium pincer complex and its active species proposed by Zhang et al.

Moreover, their study investigated the reaction mechanism using nuclear magnetic resonance (NMR) analysis. This analysis revealed the formation of complex **15**, confirmed by ^31^P-NMR, which displayed a new singlet at δ = 55.71 ppm, along with the disappearance of the starting material’s peak. Complex **15** was then converted into the active dihydride species under a hydrogen atmosphere and, in the presence of a base, resulted in the formation of dihydride complex **16**. The formation of this complex was confirmed by both ^1^H- and ^31^P-NMR, with weak signals appearing below 0 ppm in the ^1^H-NMR, and a new signal was generated in the ^31^P-NMR (Figure 8).

### 2.2. Asymmetric Transfer Hydrogenation 

**Figure 9:**
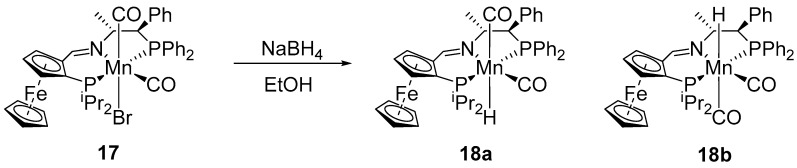
Chemical structures of [Mn(PNP’)(Br)(CO)_2_] and [Mn(PNP’)(H)(CO)_2_], as developed by Kirchner and co-workers.

In recent decades, asymmetric transfer hydrogenation (ATH), which utilizes reducing agents like isopropanol (^i^PrOH) or sodium formate (HCOONa) as hydrogen sources, has gained popularity due to its readily available hydrogen supply and operational simplicity [44,45,46,47]. In 2017, Zirakzadeh and Kirchner developed the first ATH of ketones using manganese complexes of unsymmetrical chiral PNP’ pincer ligands, achieving high conversion and enantioselectivities (up to 96% and 86% ee, respectively) in the formation of the corresponding chiral alcohols [48]. These manganese catalysts were analogues of an earlier chiral PNP’-Fe pincer complex (**3**), which featured a chiral ferrocene aliphatic scaffold and had previously been developed and tested for the asymmetric hydrogenation of ketones [32]. In their study, they successfully prepared and isolated a major isomer of bis-carbonyl complex [Mn(PNP’)(Br)(CO)_2_] (**17**), obtained by treating Mn(CO)_5_Br with the PNP’ pincer ligand. This complex was then treated with NaBH_4_ in ethanol, resulting in the formation of two inseparable isomeric bis-carbonyl Mn (I) hydride species of the type [Mn(PNP’)(H)(CO)_2_] (**18a** and **18b**, Figure 9) which served as key intermediates. All complexes were tested in the ATH of ketones, yielding comparable conversions and enantioselectivities. The ATH was conducted under mild conditions at room temperature using 4 mol% of ^t^BuOK as the base and ^i^PrOH as the hydrogen source and solvent, with a catalyst loading of 1 mol%. The absolute configuration of all reported products was determined to be (*S*); however, their catalytic efficiency was very low (turnover number (TON) ~200). 

**Figure 10:**
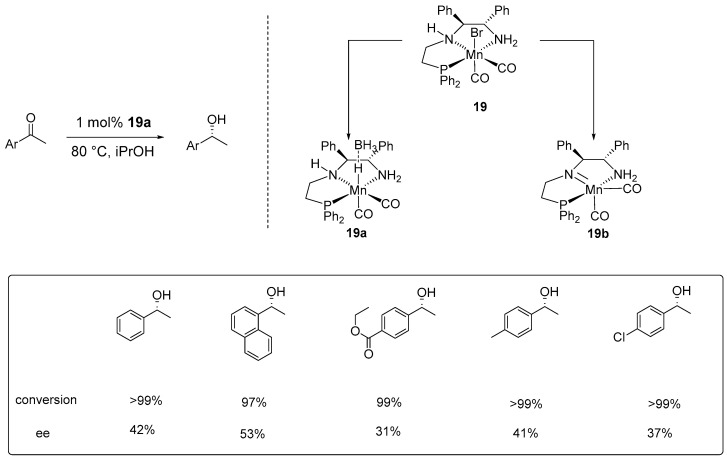
Chemical structures of manganese complexes and their activities in ATH catalytic reaction, as developed by Demmans et al.

Later, Demmans et al. synthesized a manganese pincer complex based on (1*S*,2*S*)-(-)-1,2-diphenylethylenediamine ((*S*,*S*)-DPEN)) skeleton (**19**, Figure 10). This complex was tested for the ATH of acetophenone in ^i^PrOH demonstrating moderate activity at 80 °C in the presence of ^t^BuOK, achieving >99% conversion with 37% ee. When the complex was treated separately with either ^t^BuOK in THF or NaBH_4_ in an ethanol–toluene mixture, both treatments produced active complexes (**19a** and **19b**, respectively, Figure 9) capable of catalyzing the ATH of acetophenone without the need for additional ^t^BuOK. Their study identified complex **19a** as the most effective manganese ATH catalyst, proficient in catalyzing the hydrogenation of various ketone substrates, including the base-sensitive substrate *p-*acetylbenzoate ethyl ether [49]. 

Wang et al. also developed a PNN-manganese (I) pincer complex featuring a chiral 5,6,7,8-tetrahydroquinolin-8-backbone with a ferrocene moiety (**20**), which enhanced rigidity compared to other pincer complexes containing a ferrocene moiety. This complex effectively catalyzed the ATH reaction of various substituted acetophenones and propiophenones, achieving quantitative conversions and excellent enantioselectivities (up to 99.9% ee) [50]. 

**Figure 11:**
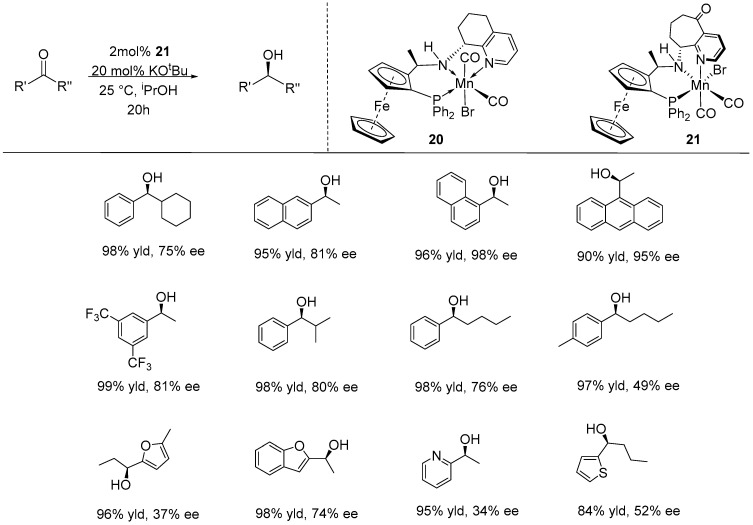
Chemical structures of catalysts **20** and **21** and their activities in ATH for selected ketones, as developed by Wang and co-workers.

Recently, the same group developed a second generation of Mn (I) catalyst, ferrocene-based, chiral PNN tridentate ligands with aliphatic cyclic groups [51,52]. Among these, catalyst **21** demonstrated the highest performance in terms of both activity and enantioselectivity during the ATH catalytic reaction. In their study, over 50 ketone substrates were tested, including phenylacetones, phenylpropanones, longer-chain alkyl-aryl ketones, and heteroaryl-alkyl substrates. Catalyst **21** demonstrated outstanding performance, with a relatively high TON of 860 and a low catalyst loading (S/C = 2000), and high enantioselectivity (up to 98% ee with (*S*)-type product) (Figure 11) [52]. Notably, most furyl-, pyridyl-, and thiophenyl alkyl ketones were compatible with the catalytic conditions and readily reduced to yield the desired alcohols with high yields (84–99%) and medium to good enantioselectivities (21–74% ee). DFT calculations revealed that the free energy barriers for hydride transfer leading to the formation of (*R*)-type chiral products with catalyst **21** were higher than those for the (*S*)-type chiral products, with a ∆∆G of 0.9–1.2 kcal/mol. The generation of the (*S*) product is favored through transition states involving an attractive aromatic π-π stacking interaction between the phenyl group of the acetophenone and the pyridyl ring of the ligand.

**Figure 12:**
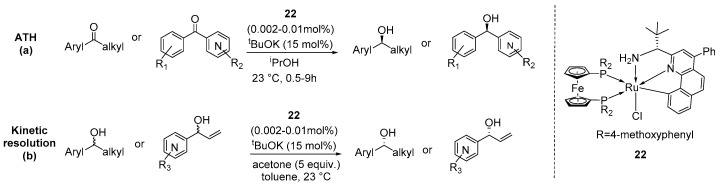
ATH catalytic reaction (**a**) and kinetic resolution (**b**) catalyzed by complex **22**, developed by Xiang and co-workers.

Xiang and co-workers designed and developed an optically pure CNN-pincer-type ruthenium complex (**22**) that exhibited extraordinarily high reactivity, achieving up to 48,000 TON and enantioselectivities of up to 99% ee in ATH across a wide range of functionalized ketones [53]. They found that a rigid tridentate ligand with a single stereogenic center, in combination with an achiral diphosphine ligand within the rationally simplified ligand structure, was sufficient to achieve high enantioselectivities for a broad array of alcohol products in ATH-based asymmetric reductions (Figure 12a). This work was inspired by Barratt’s robust [RuCl(CNN)(JOSIPHOS)] pincer complexes (JOSIPHOS = 1-[1-dicyclohexylphosphano)ethyl]-2-(diarylphophano)ferrocene), known for their remarkable activity in the AH of ketones [54,55]. The catalytic reactions were conducted using 0.002–0.01 mol% of catalyst **22**, 15 mol% of ^t^BuOK, and in the presence of ^i^PrOH at 23 °C. Various ketone substrates were successfully reduced under these conditions, including ortho- and meta-substituted aryl methyl ketones, 2- and 3- and 4-pyridyl substituted ketones, affording the corresponding alcohols with high enantioselectivities. Additionally, the same catalyst proved to be highly effective in hydrogen transfer-based kinetic resolutions of racemic aryl alkyl alcohols and heteroaryl vinyl alcohols, using acetone as the hydrogen acceptor. All tested alcohol substrates were resolved with good enantioselectivities (83–95% ee) and at practically useful conversions (Figure 12b). 

## 3. Enantioselective Hydrogenation of C=N Bonds

Chiral amines are essential building blocks for a wide range of bioactive molecules and are privileged structural motifs commonly found in pharmaceuticals and agrochemicals [56]. Over the past few decades, numerous efficient and practical methods have been developed for the synthesis of chiral amines [57]. Among these, the AH of imines (C=N) stands out as one of the most potent and atom-economical approaches for preparing chiral amines, particularly with a focus on transition metal-catalyzed AH of imines. While most efforts have traditionally focused on chiral bidentate ligands, recent advancements with chiral tridentate pincer-type ligands have shown promising progress [58]. 

**Figure 13:**
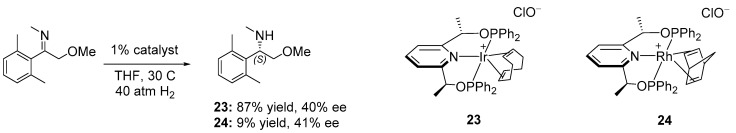
PNP-iridium and -rhodium pincer catalysts, as developed by Osborn.

Compared to the AH of ketones, progress in the hydrogenation of imines has been slower, primarily due to the relatively low reactivity of the substrates. Additionally, the amine product can coordinate with the metal catalyst as it accumulates during the AH process, inhibiting further catalysis by blocking dihydrogen coordination. Osborn and co-workers were the first to develop chiral PNP-iridium (I) and -rhodium (I) pincer complexes (**23** and **24**, respectively) and to test their efficacy in the AH of limited imine compounds [59]. Catalyst **23** achieved an 87% yield with modest enantioselectivity (40% ee). However, when the same catalyst was formed in situ, it resulted in higher enantioselectivity (55% ee) but a significantly lower yield (31%). In comparison, the analogous rhodium catalyst **24** provided 41% ee with a yield of less than 10% (Figure 13).

**Figure 14:**
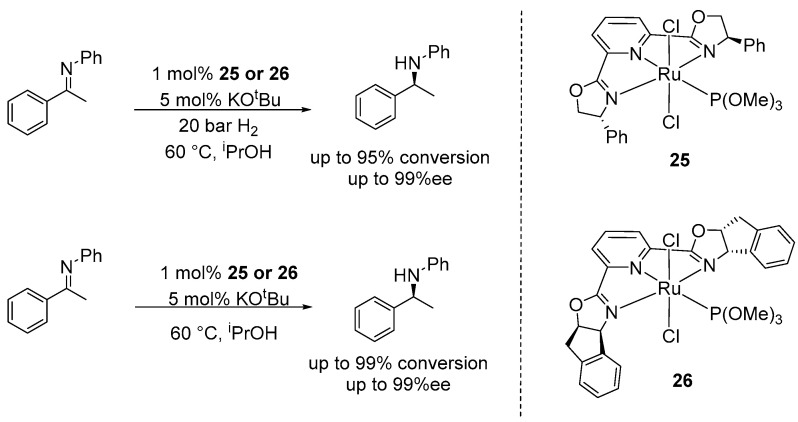
NNN-ruthenium complexes for the AH and ATH of prochiral imines, as developed by Gamasa and Pizzano.

In 2010, Kempe and co-workers developed a series of PNN-rhodium (I) pincer catalysts incorporated chiral imidazo [1,5-b]pyridazine substituted amino alcohols, achieving up to 99% conversion and 91% ee in the AH of simple imines [60]. Following this, Gamasa and Pizzano developed several NNN-Ru pincer complexes bearing chiral 2,6-bis(oxazoline)pyridine and monodentate phosphite ligands, which demonstrated high efficiency in both AH and ATH of N-aryl imines (**25** and **26**), achieving 95% conversion and up to 99% ee (Figure 14) [61]. 

**Figure 15:**
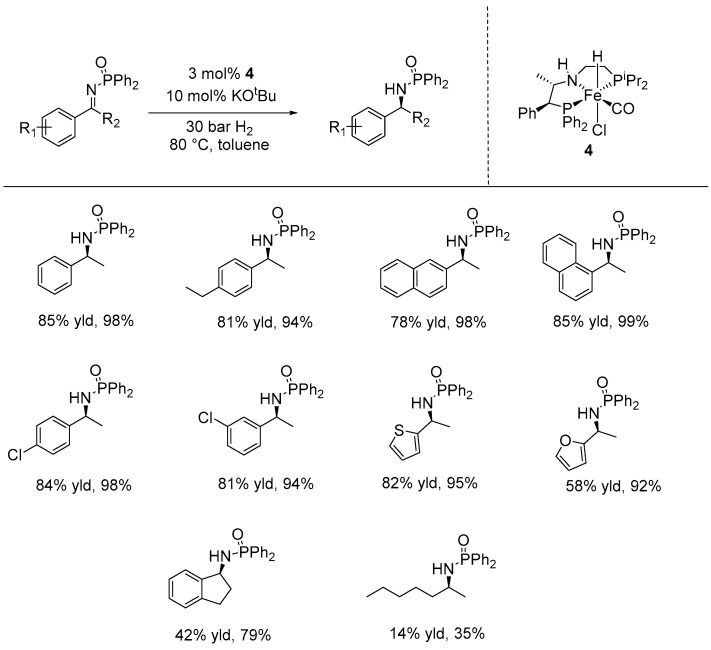
Iron-catalyzed AH of N-diphenylphophinoyl ketimines, as developed by Morris.

In 2019, Morris and co-workers employed the well-defined base-metal precatalyst, PNP’-Fe (**4**), for the AH of various prochiral imines activated with *N-*phosphinoyl or *N-*tosyl groups. This catalyst incorporates two phenyl groups on the ligand backbone, which lock the five-membered ring (*S,S*)-PPh_2_CHPhCHPhNFe into a rigid, chiral arrangement that interacts effectively with the imine substrate. The catalytic reactions were conducted using 3 mol% of catalyst **4** and 10 mol% of KO^t^Bu under 30 bar hydrogen gas at 80 °C in toluene, yielding the corresponding amines with up to 85% yield and enantioselectivities of up to 99% ee. A variety of *N-*phosphinoyl substrates were well-tolerated under these catalytic conditions, including aryl alkyl ketones with electron-withdrawing and electron-donating groups, as well as oxygen- or sulfur-containing heterocycles substituted *N-*phosphinoylimines (Figure 15) [62].

**Figure 16:**
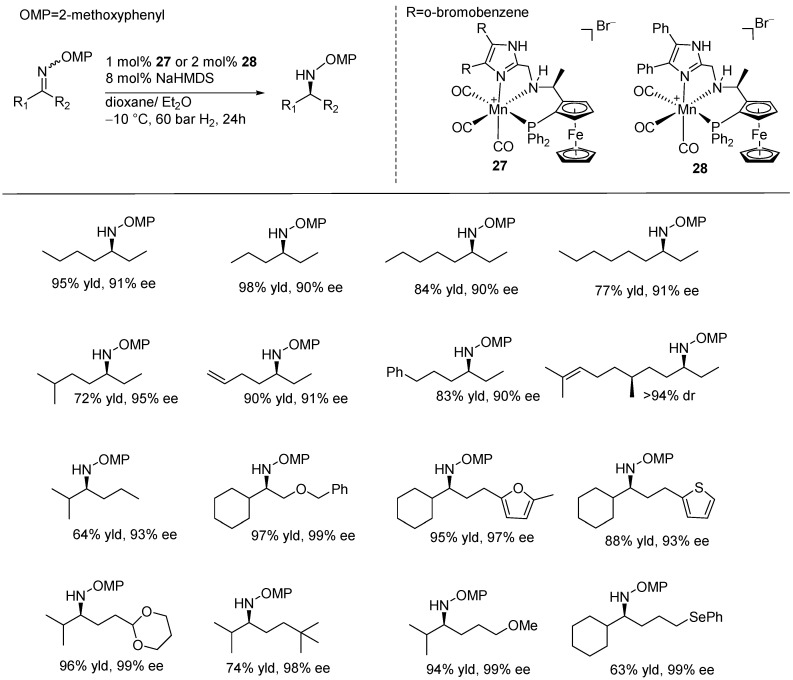
Mn-catalyzed AH for selected substrates of imines with various alkyl groups, as developed by Lan and Liu.

Recently, Lan and Liu introduced a privileged class of manganese catalysts for the AH of dialkyl ketimines that gave a range of chiral amine products. In their study, they synthesized various manganese NNP-pincer complexes featuring 4,5-disubstituted imidazole ligands with a ferrocene unit, incorporating the chiral stereogenic center into the N-dialkyl backbone. Among these, the catalyst featuring an o-bromophenyl group (**27**) was found to be the most effective. The catalytic reactions were carried out using 1 mol% of this catalyst, with sodium bis(trimethylsilyl)amide (NaHMDS) as the base, under 60 bar of hydrogen at −10 °C in a dioxane-diethyl ether solution. The desired products were obtained with good yields (72–98%) and excellent enantioselectivities (90–95% ee), achieving a TON up to 107,800 (Figure 15). Additionally, satisfactory results were achieved by using the diphenyl-substituted complex (**28**) as the precatalyst. The catalytic reactions, performed using 2 mol% of catalysts, with 10 mol% of NaO^t^Bu in dioxane at 60 °C under 60 bars H_2_, produced good to excellent yields (63–99%) and high levels of enantioselectivity (92–99% ee) across substrates bearing various functional groups, such as alkenyl, alkynyl, phenyl, selenyl, benzyloxy, thienyl, furyl, t-butyl, 1,3-diohexyl-2-yl and methoxy groups (Figure 16) [63].

## 4. Enantioselective Hydrogenation of C=C Bonds

The hydrogenation of C=C double bonds has long been a focal point for chemists. A significant breakthrough in homogeneous hydrogenation was achieved by Wilkinson in the 1960s with the development of a rhodium catalyst, Rh(PPh_3_)_3_Cl, commonly known as “Wilkinson’s complex” [64]. This pioneering work paved the way for the practical asymmetric hydrogenation of C=C double bonds using precious metal complexes, such as those of ruthenium, iridium, and osmium, in combination with chiral ligands. In 1968, Knowles and Horner were the first to report asymmetric alkene hydrogenation using transition-metal complexes [65,66,67]. Pro-chiral alkenes can be broadly classified into unfunctionalized (or non-coordinating) and functionalized (or coordinating) alkenes. The enantioselective reduction of unfunctionalized alkenes is particularly challenging due to the absence of suitable directing groups. In contrast, functionalized alkenes, which contain coordinating atoms and directing groups (such as NHCOR, COOR, B(OR)_2_), facilitate catalyst-substrate complex formation, leading to high selectivity in hydrogenation reactions. 

**Figure 17:**
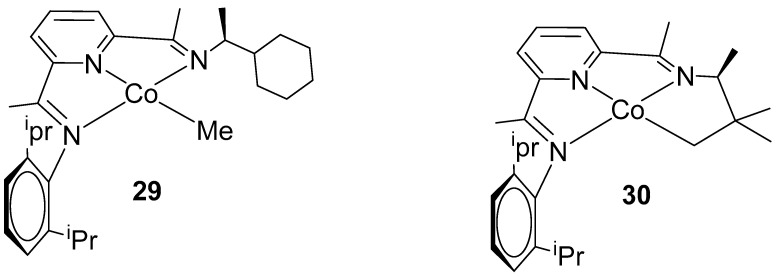
NNN-Co pincer complex-based bis(imino)pyridine skeleton, as developed by Chirik.

Chirik and co-workers made significant progress in the enantioselective hydrogenation of alkenes utilizing pincer-type complexes. In 2012, they designed and synthesized an NNN-Cobalt-based bis-(imino)pyridine skeleton featuring a single enantiomer of chiral alkyl amine, inspired by the innovative study of Bianchini and co-workers [68]. By treating the bis(imino)pyridine cobalt dichloride complexes with NaBEt_3_H, they produced the corresponding cobalt monochloride, which was then methylated with MeLi to yield the active catalysts **29** and **30** (Figure 17) [69]. 

**Figure 18:**
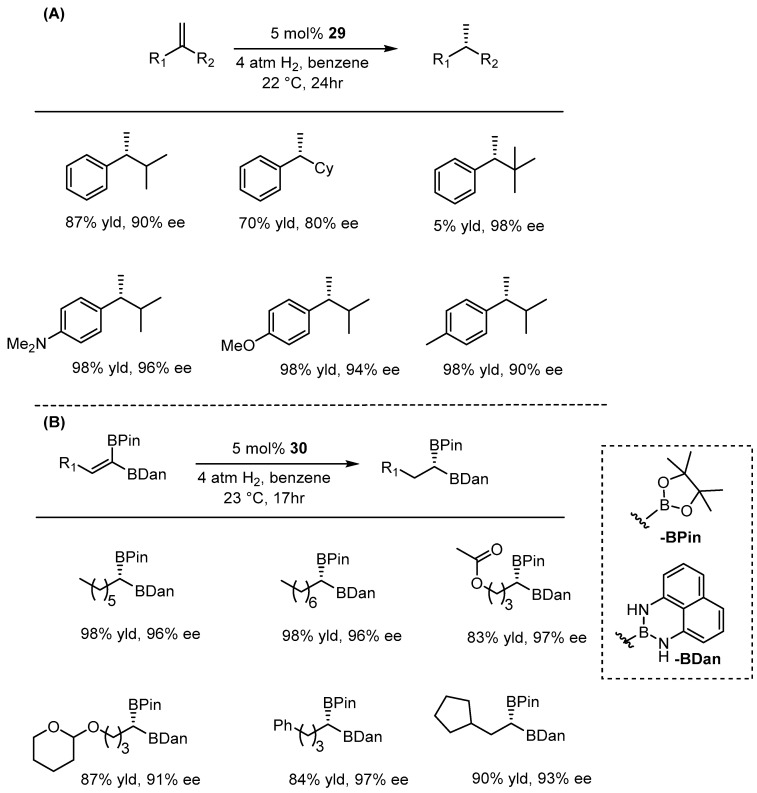
Cobalt-catalyzed AH of unfunctionalized alkenes (**A**) and 1,1-diboryl alkenes (**B**).

They utilized catalyst **29** in the asymmetric hydrogenation of unfunctionalized alkenes, achieving conversions of up to 98% and enantioselectivities of up to 98% ee (Figure 18A). Additionally, the same catalyst was effective for the hydrogenation of cyclic alkenes, providing up to 97% conversion and 99% ee [70]. In 2020, the same research group employed catalyst 30 for the asymmetric hydrogenation of unsymmetric 1,1-diboryl alkenes. Using 1 mol% of catalyst **30** and 4 atm of H_2_ gas, the substrates were hydrogenated into the corresponding 1,1-diboryl alkanes, achieving up to 98% conversion and 98% ee, favoring the (*S*) enantiomer (Figure 18B) [71]. It is noteworthy that this catalyst showed no activity for the hydrogenation of 1,1-disubstituted styrene derivatives; only the boron-substituted alkene was hydrogenated, indicating an activating effect of the boron groups, yielding good results.

**Figure 19:**
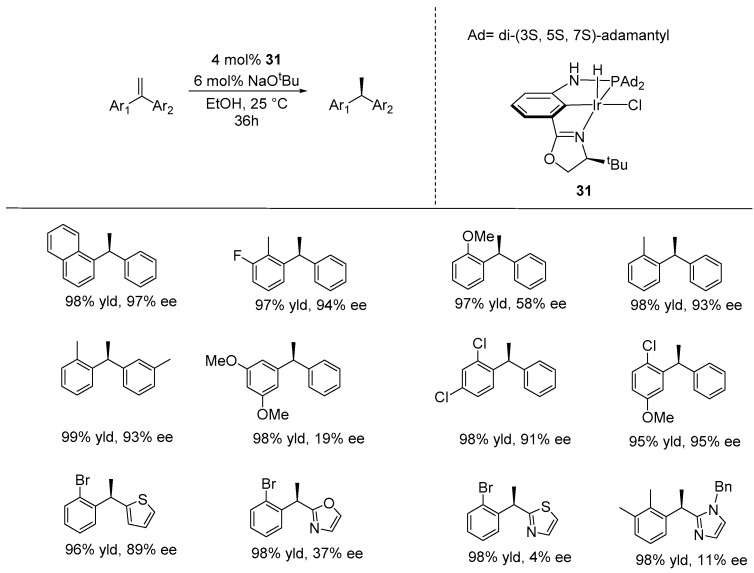
Iridium-catalyzed TAH of 1,1-diarylalkenes, as developed by Huang.

Subsequently, Huang and co-workers developed chiral iridium complexes ligated by anionic oxazoline-bearing PCN_ox_ pincer-type ligands, which they employed in the ATH of diarylethenes, using ethanol as a benign hydrogen donor and solvent. Notably, PCN_ox_ pincer complexes overcome the tendency for carbonylation, making them suitable for catalytic ATH of inactivated alkenes with EtOH [72]. This contrasts the classic bis(phosphine)-based (PCP)-Ir complexes, which quickly undergo EtOH decarbonylation, leading to the formation of catalytically inactive carbonyl species [73,74]. They found that incorporating the bulky di-(3*S*, 5*S*, 7*S*)-adamantyl (Ad) groups on the phosphorus atom produced the most selective catalyst in their series, yielding chiral products with up to 98% yield and 96% enantioselectivity. A wide range of substrates underwent mild hydrogenation to form 1,1-diarylethanes with high enantioselectivities (using 4 mol% of catalyst **31**, 6 mol% of NaOtBu, and ethanol), although further improvement is needed for some heteroaryl-substituted ethenes (Figure 19) [75]. 

**Figure 20:**
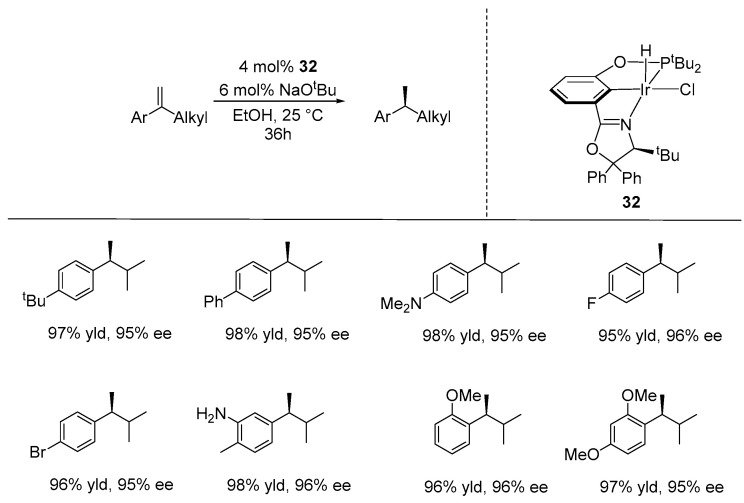
NCP-Ir-catalyzed ATH of 1-aryl-1-alkylethenes, as developed by Huang.

Following this significant progress, the same research group, over the past two years, developed the ATH of 1-aryl-1-alkylethenes and 1,1-dialkylethenes with ethanol [76,77]. To improve the enantioselectivity of the ATH of alkylstyrenes, they carried out structural modifications on their previous chiral PCN_ox_-pincer ligands. Thus, they designed and synthesized a PCN_ox_-Ir pincer complex containing geminal phenyl groups on the oxazoline ring (**32**), which was applied to the ATH of 1-aryl-1-alkylehtenes in the presence of 4 mol% of catalyst, 6 mol% of NaO^t^Bu, and EtOH as the hydrogen donor and solvent, yielding up to 98% and 98% ee (Figure 20) [76]. 

**Figure 21:**
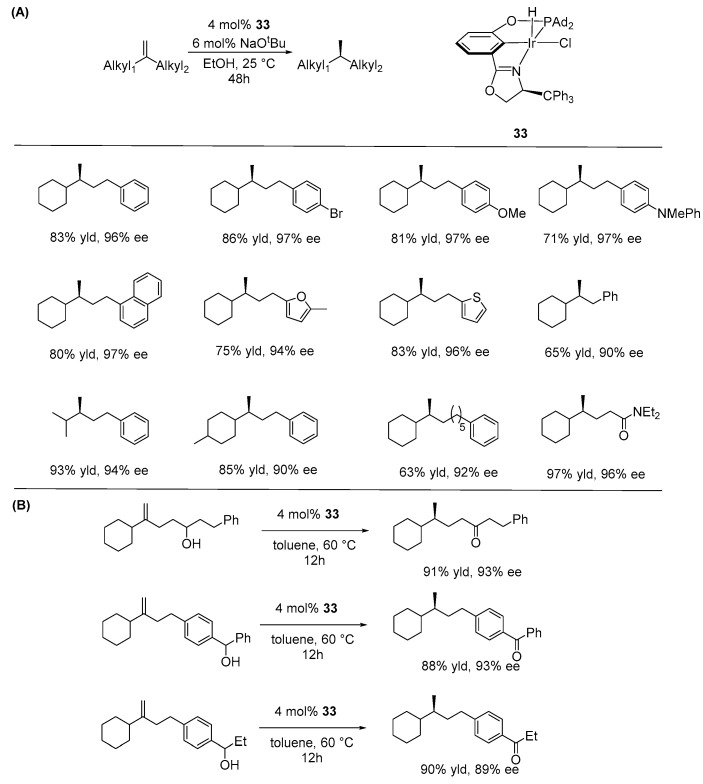
NCP-Ir-catalyzed ATH of 1,1-dialkylethenes (**A**) and redox isomerization of disubstituted alkenols (**B**), as developed by Huang.

Moreover, they synthesized a new iridium catalyst bearing bulky adamantyl groups at the P atom and a trityl group on the oxazoline (**33**). They proposed that using a bulky pincer ligand helps minimize isomerization activity, which likely plays a crucial role in achieving high enantioselectivity in the asymmetric hydrogenation of 1,1-dialkylethenes, a particularly challenging class of alkenes. As a result, they demonstrated that this catalyst enabled a highly enantioselective reduction of 1,1-dialkylethenes across a broad range of substrates. The catalyst also showed tolerance toward various substitutions on the aryl group, including halides, ethers, and amines, as well as 2-furanyl, 2-thiophenyl, and 1-naphthyl groups (Figure 21A). Interestingly, the same catalyst was applied to the enantioselective conversion of disubstituted alkenols into chiral carbonyls without an external H-donor. This formally redox-neutral reaction resembles the asymmetric chain-walking isomerization of alkenols [78]. Notably, their novel protocol enables the construction of trialkyl-substituted tertiary carbon stereocenters, a capability demonstrated for the first time in their work (Figure 21B).

## 5. Enantioselective Hydrophosphination

**Figure 22:**
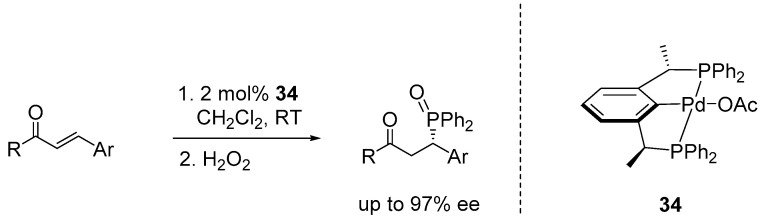
Asymmetric hydrophosphination catalyzed by chiral PCP-Pd pincer complex, developed by Duan and Zhang.

Organophosphorus molecules are an important class of compounds extensively used as transition-metal ligands and organocatalysts in a variety of catalytic transformations [79,80]. Beyond their catalytic roles, they are also valuable in pharmaceuticals, materials science, and agrochemicals [81,82,83]. Transition metals homogeneously catalyze the addition of a P-H bond across a C=C bond, offering an atom-efficient method for synthesizing phosphine-based compounds [84]. Asymmetric phosphine compounds are of significant interest due to their crucial role in developing novel transition metal-catalyzed asymmetric transformations. Duan and co-workers made notable contributions by utilizing the chiral PCP-palladium pincer complex (**34**), previously developed by Zhang [85], to facilitate the asymmetric diarylphosphonation of β-substituted enones. The addition reaction of Ph_2_PH to enones, in the presence of 2 mol% of **34**, followed by successive oxidation with H_2_O_2_, resulted in chiral phosphine oxide products with good yields and excellent enantioselectivities, achieving up to 97% ee (Figure 22) [86].

Significant progress has since been made in developing asymmetric hydrophosphination, including hydrophosphination of nitroalkanes, conjugate addition of *β*,*γ*-unsaturated *α-*keto esters, and the conjugate addition of *α*,*β*,*γ*,*δ*-unsaturated carbonyl derivatives [87,88,89,90,91]. 

**Figure 23:**
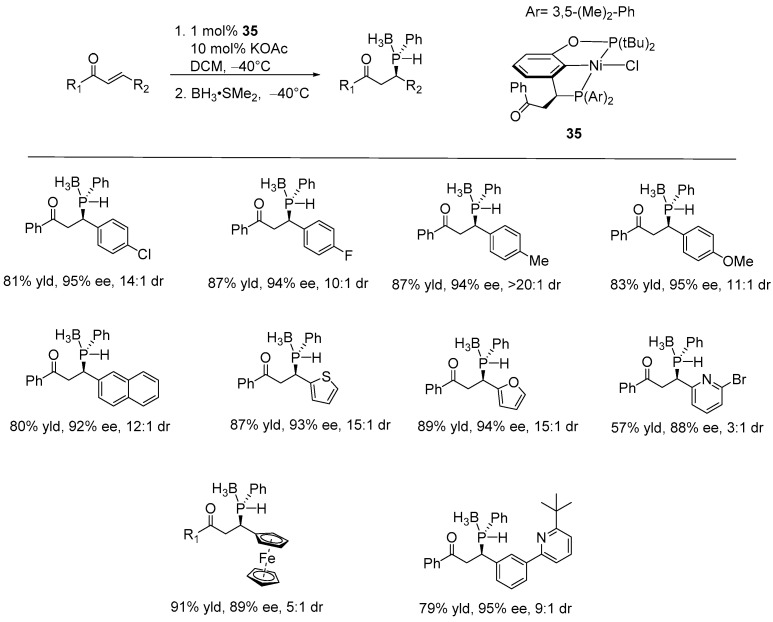
PCP’-nickel-catalyzed asymmetric hydrophosphination of enones, as developed by Duan.

Later, Duan and co-workers developed the first example of a direct catalytic asymmetric strategy for constructing P-stereogenic secondary phosphine-borane using their newly developed unsymmetric bisphosphine PCP’-nickel pincer complex (**35**). Notably, the resulting chiral secondary phosphines are valuable precursors that can be readily converted into other phosphorus compounds, serving as chiral phosphine ligands for asymmetric catalysis. The asymmetric hydrophosphination of a wide variety of enones with H_2_PPh, followed by the addition of BH_3_, resulted in good yields (57–92%) with excellent enantio- and diastereoselectivity (dr) (up to 99%, >20:1 dr). This method tolerated both electron-withdrawing and electron-donating substituents attached to *β-*aryl *α,β-*unsaturated phenyl ketone substrates, as well as, *β-*naphthyl and *β-*ferrocenyl, *β-*furyl, *β-*thinyl, and *β-*pyridyl enones (Figure 23) [92]. Two years later, the same research group reported the development of an unsymmetric PCP’-nickel pincer complex and its application in the asymmetric addition of phosphine to electron-deficient alkenes. This method yielded various chiral phosphines in 61–92% yields with enantioselectivities of up to 98% ee. The catalytic reaction was carried out with 2 mol% of the catalyst in the presence of 10 mol% of KOAc as the base, in acetonitrile at −20 °C, followed by oxidation with excess H_2_O_2_ [93]. 

Moreover, catalyst **35** was successfully utilized in the asymmetric addition of primary phosphines to azo compounds, yielding P-stereogenic phosphanyl hydrazine products with up to 98% yields and 96% ee. They further demonstrated that the P-H and P-N bonds in these phosphanyl hydrazine building blocks could react sequentially and stereospecifically, allowing for the synthesis of various P-stereogenic compounds with significant structural diversity. Thus, the obtained P-stereogenic secondary phosphine-boranes can be directly converted into chiral tertiary amino-phosphine-boranes by its reaction with alkyl halides in the presence of bis(trimethylsilyl)amide at −78 °C. Alternatively, nucleophilic substitution accompanied by P-N bond cleavage can convert these compounds into alkoxy-substituted tertiary phosphine-boranes through an acid-mediated stereospecific replacement of the diazinyl group with alcohols, achieving products with high enantioselectivities [94]. 

**Figure 24:**
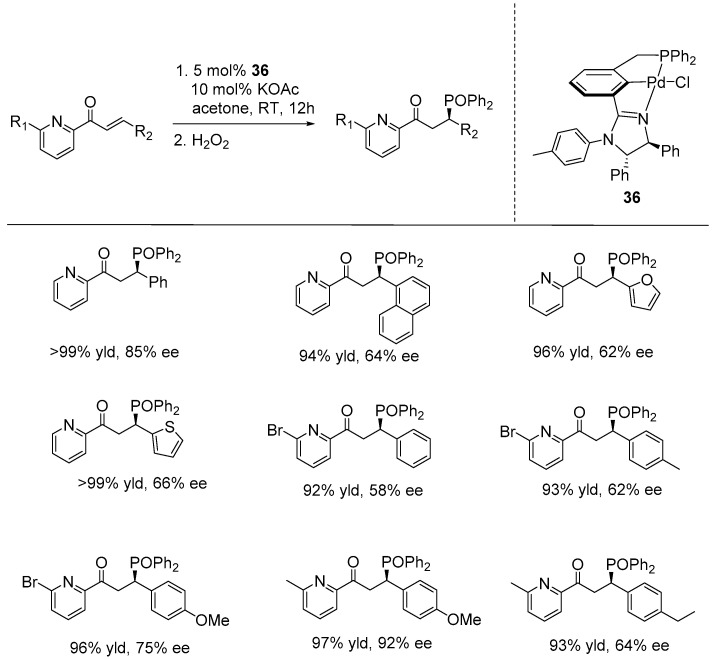
PCN-Pd-catalyzed asymmetric hydrophosphination of 2-alkenoylpyridine, as developed by Gong and Song.

In 2022, Gong and Song synthesized a series of chiral PCN-Pd pincer complexes featuring aryl-based (phosphine)-(imidazoline) ligands. Among these, complex **36** demonstrated the best stereocontrol in the catalytic asymmetric hydrophosphination of 2-alkenoylpyridines. Using catalyst **36**, along with KOAc as the base, the reaction of various 2-alkenoylpyridines with Ph_2_PH proceeded smoothly in acetone at room temperature, yielding structurally diverse optically active phosphine derivatives in excellent yields (>99%) and with moderate to good enantioselectivities, reaching up to 85% ee (Figure 24) [95]. 

**Figure 25:**
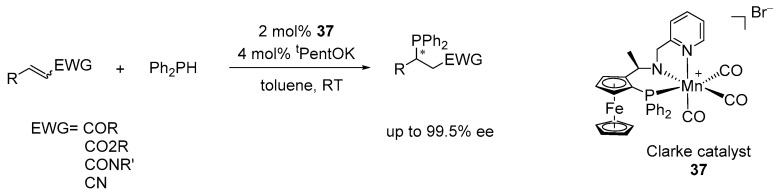
NNP-Mn-catalyzed hydrophosphination of α,β-unsaturated compounds. * indicates chiral center.

Additionally, Harutyunyan and co-workers demonstrated the use of a chiral NNP-Mn pincer catalyst, originally developed by Clarke (catalyst **37**) [26], in the asymmetric hydrophosphination of various Michael acceptors, including α,β-unsaturated carbonyl derivatives of ketones, esters, nitriles, and carboxamides. This reaction allows for the formation of a range of phosphine-containing chiral products, achieved through H–P bond activation via metal-ligand cooperation [16,96]. In their studies, they used 2 mol% of catalyst **37**, 4 mol% of ^t^PentOK, and toluene as the solvent at room temperature, employing diphenylphosphine and α,β-unsaturated compounds as the reactants, which afforded the corresponding chiral phosphines with moderate to high enantioselectivity (Figure 25).

## 6. Enantioselective Alkynylation 

The enantioselective alkynylation of ketones is recognized for the synthesis of chiral propargylic alcohols. Typically, the introduction of an alkynyl group into a carbonyl compound is achieved through the in situ generation of metal alkynyl intermediates from terminal alkynes using stoichiometric amounts of metals like lithium or zinc [97,98,99,100,101]. However, from an atom-economy perspective, the direct activation of alkynes by an organometallic catalyst is a more desirable and efficient method. Several highly effective and enantioselective direct alkynylation reactions have been reported, including those utilizing chiral pincer catalysts [102,103]. Oshima and Mashima were the first to demonstrate the high performance of phebox-rhodium pincer complexes in the direct enantioselective alkynylation of ketones. In their study, they successfully carried out the asymmetric alkynylation of *α*-ketoesters with various aryl- and alkyl-substituted terminal alkynes, yielding the corresponding chiral tertiary propargylic alcohols with ee of up to 99% [104]. They later extended this methodology to the direct asymmetric alkynylation of *α*-ketiminoesters, resulting in the formation of propargylic amine derivatives [105]. 

**Figure 26:**
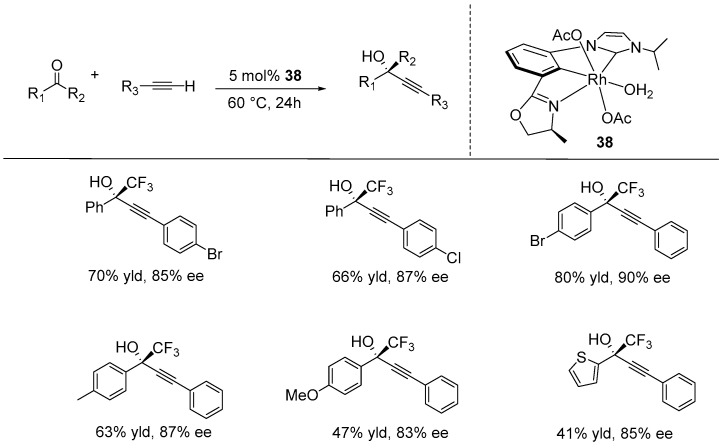
Asymmetric direct alkynylation of activated ketones catalyzed by CCN-Rh acetate complexes, as developed by Nishiyama.

Nishiyama and co-workers developed CCN-Rh pincer complexes containing N-heterocyclic carbene and oxazoline ligands and utilized them in the asymmetric alkynylation of activated ketones. The catalytic reactions of fluoroalkyl-substituted ketones (using 5 mol% of catalyst **38** at 60 °C for 24 h), including aryl-COCF_2_X (X = F, Cl, H), with aromatic and aliphatic alkynes, produced the corresponding chiral propargylic alcohols with high enantioselectivities of up to 93% (Figure 26) [106].

**Figure 27:**
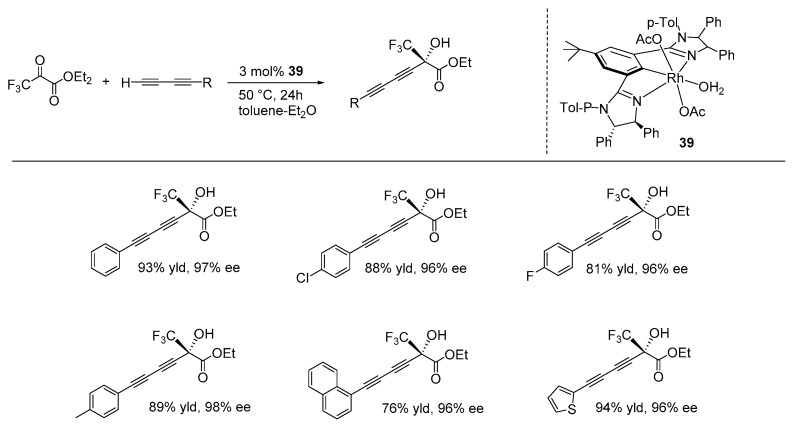
NCN-rhodium-catalyzed direct enantioselective alkynylation of trifluoropyruvates with terminal 1,3-diynes, as developed by Song and Gong.

In 2023, Song and Gong described the synthesis of NCN-Rh pincer complexes with bis(imidazolinyl)phenyl ligands. They utilized these complexes in a direct catalytic enantioselective alkynylation reaction of trifluoropyruvates with terminal 1,3-diynes, resulting in the production of optically active trifluoromethylated tertiary alcohols featuring the diyne moiety. The reaction was performed under mild conditions, using 3 mol% of catalyst **39** at 50 °C with a toluene-diethyl ether solvent mixture. The reaction tolerated various functional groups, including F^−^, Cl^−^, Br^−^, CH_3_^−^, CH_3_O^−^, thiophene, and naphthalene on the diyne, with ee values up to 95% (Figure 27) [107].

## 7. Miscellaneous Enantioselective Reactions 

**Figure 28:**
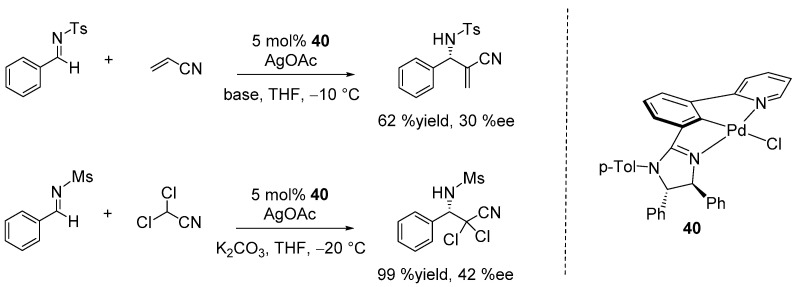
Asymmetric Aza–Morita–Baylis–Hillman reaction of acrylonitrile and dichloroacetonitrile with N-methanesulfonylimine catalyzed by NCN-Pd pincer complexes.

In 2022, Song and Gong synthesized a series of chiral NCN’-Pd pincer complexes incorporating chiral 1-(2-imidazolinyl)-3-(2-pyridyl)-phenyl ligands. These catalysts were employed in the asymmetric Aza–Morita–Baylis–Hillman reaction of acrylonitrile with various N-toluenesulfonylimines (Ts-imines) as well as reactions of dichloroacetonitrile with N-methanesulfonylimines (Ms-imines), achieving moderate enantioselectivities (up to 44% ee). The optimal catalysts identified in their study featured Pd pincers with both pyridine N-donor and (4*S*,5*S*)-diphenyl substituted imidazoline (catalyst **40**) (Figure 28) [108].

The same research group reported the asymmetric carbenoid C-H insertion of 3-diazooxindoles into 1,4-cyclohexadiene using a chiral bis(imidazole) NCN-Ir pincer complexes, analogous to rhodium complex **38**. These catalytic reactions, conducted at 0.5 mol% of catalyst, at 0 °C, tolerated various functional groups, including phenyl rings substituted with both electron-withdrawing and electron-donating groups. The process yielded a range of a chiral 3-substituted oxindoles in good yields, with enantioselectivities ranging from moderate to excellent, achieving up to 99% ee [109]. 

**Figure 29:**
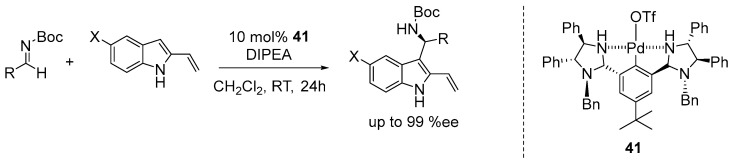
Asymmetric reaction of 2-vinylindole to N-Boc imines catalyzed by NCN-Pd pincer complex.

Arai and co-workers described the synthesis of a chiral imidazolidine-containing NCN-pincer Pd-OTf (Tf = trifluoromethane sulfonate or triflate) complex, which was utilized in the catalytic asymmetric Friedel−Crafts reaction of 2-vinylindoles to *N-*Boc imines. The catalytic reactions were performed under mildly basic conditions using diisopropylethylamine amine (DIPEA) as the base and in the presence of 10 mol% of catalyst **41** in dichloromethane at room temperature. The catalytic reaction exhibited good tolerance for benzaldehyde-derived *N-*Boc imines with both electron-withdrawing and electron-donating groups at the *ortho-*, *meta-*, and *para-* positions of the benzene ring, affording chiral products with high yields and enantioselectivities (up to 99% ee) (Figure 29) [110].

**Figure 30:**
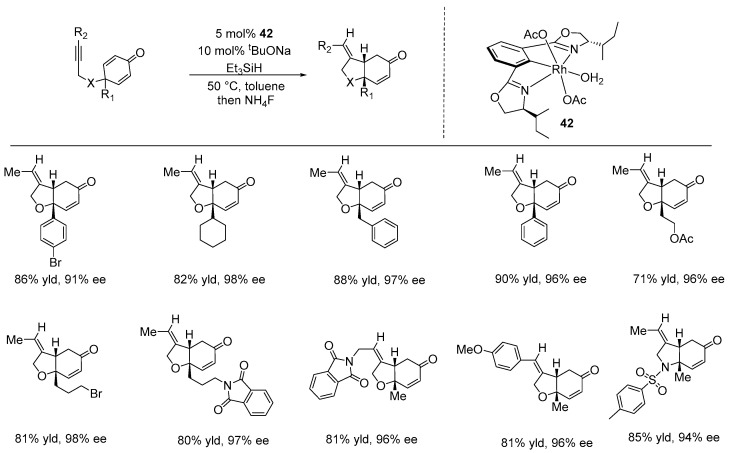
NCN-rhodium-catalyzed enantioselective reductive cyclization of alkynyl-tethered cyclohexadienones, as developed by Tan and Tian.

Recently, Tan and Tian developed a novel method for the enantioselective reductive cyclization of alkynyl-tethered cyclohexadienones (1,6-enynes) using an NCN-Rh pincer catalyst bearing (2,6-bisoxazolinylphenyl) ligands, originally developed by Nishiyama and co-workers [111,112,113]. Catalytic asymmetric cascade cyclization reactions of 1,n-bifunctional molecules, particularly 1,6-enynes, are recognized as important and efficient methods for constructing enantioenriched cyclic compounds [114,115,116]. However, this reaction poses significant challenges, primarily due to the difficulty in controlling chemoselectivity between the enones and alkynes, the regioselectivity of the alkyne’s insertion, and the potential side reactions (such as alkyne polymerization). Their study describes a catalytic system for the enantioselective reductive cyclization of alkynyl-thethered cyclohexadienones, using 5 mol% of an NCN-Rh pincer complex **42**, along with the addition of 10 mol% of ^t^BuONa and triethyl silane (Et_3_SiH). The method resulted in the formation of cis-hydrobenzofurans and cis-hydroindoles with high enantioselectivities (up to 99% ee), demonstrating compatibility with a variety of functional groups, including halogen, silyl-protected alcohol, ester, imide, acetal, ketone, and amide (Figure 30) [117].

**Figure 31:**
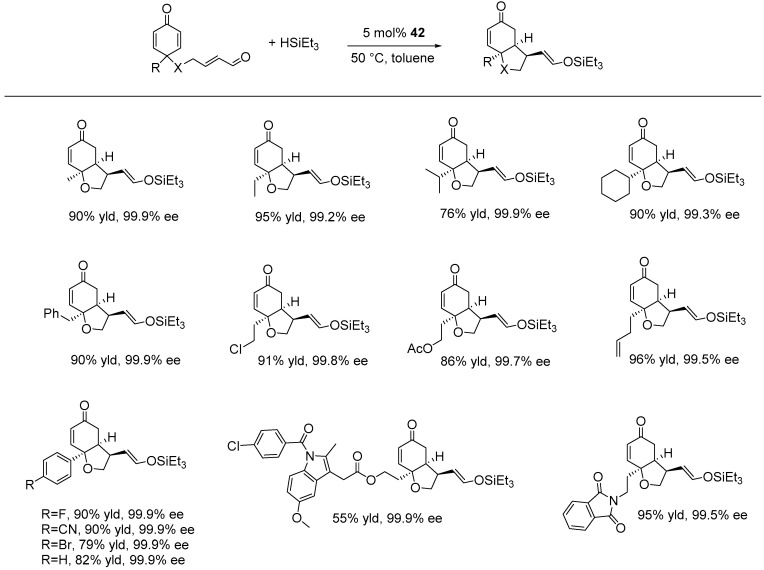
NCN-rhodium-catalyzed enantioselective hydrosilylation/cyclization of cyclohexadienone-tethered α,β-unsaturated aldehydes, as developed by Tian.

In the same year, the same research group reported a method for the enantioselective hydrosilylation/cyclization of cyclohexadienone-tethered α,β-unsaturated aldehydes (1,6-dienes) using triethylsilane. This process resulted in the formation of various cis-hydrobenzofurans, cis-hydroindoles, and cis-hydroindenes bearing silyl enol ethers. The reactions were catalyzed by 5 mol% of **42** in toluene at 50 °C, achieving good to excellent yields and high enantioselectivities (up to 90% yield and 99% ee). Aryl substituents, including phenyl, p-FC_6_H_4_, p-BrC_6_H_4_, and p-CNC_6_H_4_, on the quaternary carbon of cyclohexadienone, were well-tolerated, producing the bicyclic products in 66–90% yield with 99.9% ee (Figure 31) [118]. Notably, the same catalyst had previously been applied in other enantioselective catalytic reactions, including the hydrosilylation of conjugated alkenes and ketones, as well as the desymmetrization of cyclohexadienones [111,119,120,121].

**Figure 32:**
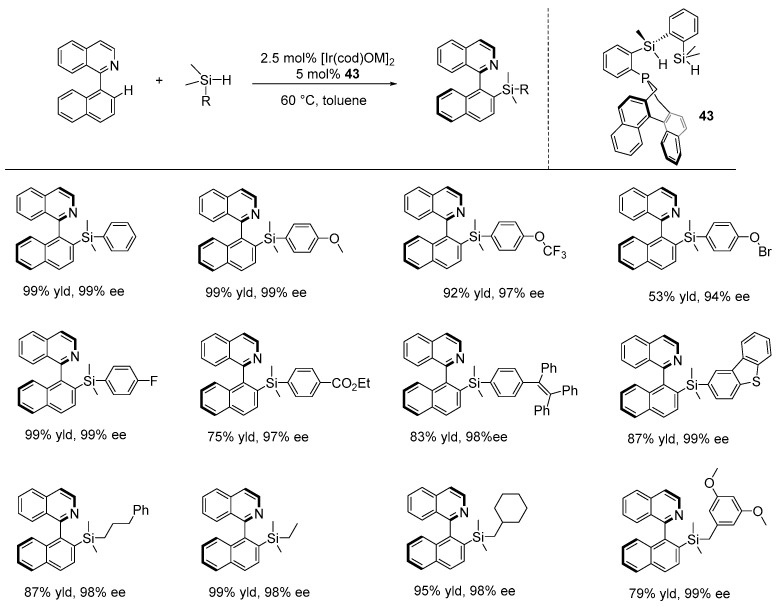
PSiSi-Ir-catalyzed atroposelective intermolecular C-H silylation reaction, as developed by He and Ge.

Interestingly, He and Ge developed a novel chiral PSiSi pincer-type ligand (**43**) that enabled a groundbreaking Ir-catalyzed atroposelective intermolecular C-H silylation reaction between 2-arylisoquinolines and hydrosilanes. Their work was inspired by the earlier achiral silyl complexes reported by Tilley [122] and Tobita [123], who demonstrated that these complexes can create an electron-rich metal center with a coordinatively unsaturated site, thereby promoting more efficient C-H bond activation through oxidative addition. In their manuscript, He and Ge demonstrated a catalytic reaction between 2-arylisoquinolines and various hydrosilanes using the in situ generation of an Ir-PSiSi catalyst (5 mol%) in toluene at 60 °C, with 3,3-dimethylbut-1-ene (TBE) as the hydrogen acceptor. X-ray crystallography confirmed the absolute configuration of the enantioenriched compounds. The reaction showed broad tolerance to various substituents on hydrosilanes, including both electron-donating and electron-withdrawing groups, producing a wide range of axially chiral silanes with high yields and excellent enantioselectivities (up to 99% yield and 99% ee). Additionally, they demonstrated that these enantioenriched silylated products can serve as versatile intermediates for the asymmetric synthesis of a variety of functionalized axially chiral compounds (Figure 32) [124].

**Figure 33:**
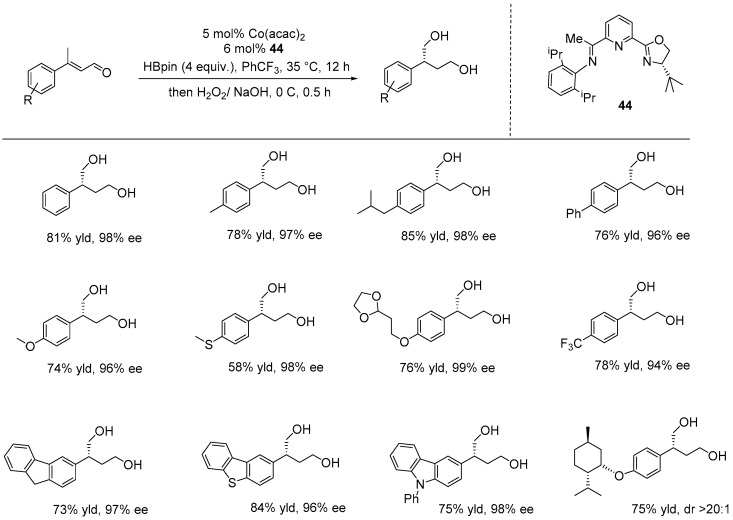
NNN-Co-catalyzed enantioselective dihydroboration of enals, as developed by Zhao.

Additionally, several researchers have developed and reported on pincer-type ligands used in the asymmetric borylation catalytic reactions, including the diboration and hydroboration of unsaturated compounds, leading to the formation of chiral borylated products. These compounds can then be stereospecifically converted into chiral alcohols and amines. For instance, Nishiyama and co-workers designed NCN-Rh acetate pincer complexes that exhibited excellent catalytic performance in the asymmetric conjugate borylation of *E*-cinnamyl esters with B_2_pin_2_, yielding β-hydroxy esters with high enantioselectivities (up to 97%). The complexes also proved effective in the asymmetric diboration of alkenes, leading to 1,2-diborane compounds, which were subsequently oxidized to produce chiral diols with outstanding enantioselectivities [11,125,126]. Recently, Zhao and his research group reported on the asymmetric dihydroboration of both carbonyl and alkene functional groups, specifically in enals. In their study, they employed an in situ generated catalyst based on cobalt and a chiral NNN ligand containing an oxazoline moiety (**44**). The catalytic reactions were performed under mild conditions using 5 mol% of the active catalyst, HBpin as the borylation reagent, in CF_3_-benzene at 30 °C. This transformation yielded chiral 1,4-borylethers with excellent enantioselectivities (up to 99% ee) and good yields (up to 85%), demonstrating good functional group tolerance and broad substrate scope. The reactions successfully addressed challenges related to chemoselectivity, regioselectivity, and enantioselectivity, as well as the issue of heteroatom elimination in transition-metal catalysis, despite numerous potential competing pathways (Figure 33) [127].

**Figure 34:**
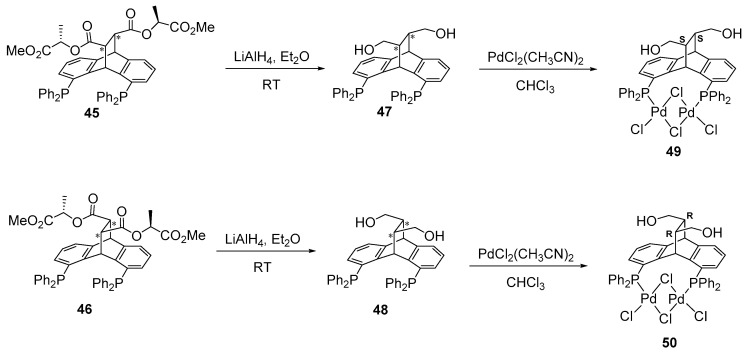
Synthetic approach to synthesizing the chiral enantiopure PC(sp^3^)P pincer ligands, as developed by Gelman.* indicates chiral center.

In 2021, Gelman and co-workers reported a privileged and straightforward synthetic approach for preparing chiral, enantiopure 3-dimensional PC(sp^3^)P pincer ligands with pendant hydroxyl functional groups. The catalyst derived from this ligand demonstrated the ability to activate and form chemical bonds through an alkoxy/alkoxide coordination switch, proving effective in various hydrogen transfer reactions [128,129,130]. Their synthetic route involved an asymmetric carbo-Diels–Alder reaction between 1,8-bis(diphenylphosphino)anthracene and enantiomerically pure bis(methyl-(*S*)-lactyl) fumarate, which resulted in the formation of a pair of separable diastereomers (**45** and **46**). These diastereomers were subsequently reduced with LiAlH_4_ at room temperature, yielding two separated enantiomers (**47** and **48**) with a 70% yield. The absolute stereochemistry of one of the enantiomers was determined to be (*S*,*S*) based on structural analysis of the crystal quasi-closed dipalladium complex (**49** and **50**) (Figure 34) [131]. Although they did not demonstrate any enantioselective reactions catalyzed by the synthesized enantiopure pincer complexes, the metaled C-chiral carbon centers have the potential to induce enantioselection and stereochemical control due to their rigid and well-defined coordination environment. Their unique 3-dimensional structure may also introduce new coordination modes, such as ligand-metal cooperative enantioselective pathways, and inspire future innovations in asymmetric catalytic transformations.

## 8. Conclusions

In this review, we discussed recent advancements in the development of chiral pincer-type ligands and their transition metal complexes, focusing on their applications in asymmetric catalytic reactions. A variety of enantioselective transformations were described, including the asymmetric (transfer) hydrogenation of both polar and non-polar bonds, hydrophosphination, alkynylation, and Friedel–Crafts reactions. Additionally, we highlighted the enantioselective reductive cyclization of alkynyl-tethered cyclohexadienones and the Aza–Morita–Baylis–Hillman reactions involving acrylonitrile and dichloroacetonitrile with *N-*methanesulfonylimine.

Chiral pincer-type ligands have proven exceptionally valuable in asymmetric catalysis due to their unique rigidity, tunability, and robustness combination. The tridentate coordination mode of these ligands creates a highly defined steric and electronic environment around the metal center, which is crucial for controlling stereoselectivity and achieving high enantioselectivity in various catalytic transformations. Incorporating stereogenic elements, such as chiral centers at benzylic positions, stereogenic phosphorus atoms, or chiral auxiliary groups, has significantly advanced their use in the synthesis of enantioenriched compounds.

The versatility of chiral pincer complexes is evident in their wide-ranging applications, spanning numerous branches of catalysis and extending into fields such as organic materials, bioinorganic chemistry, and supramolecular chemistry. Their structural tunability allows for the customization of catalytic properties to suit specific reactions, making them adaptable tools for constructing complex, enantioenriched molecules.

Looking ahead, the continued development of novel chiral pincer ligands and their corresponding metal complexes holds great promise for advancing asymmetric synthesis. Innovations in ligand design, including the incorporation of new stereogenic elements and the exploration of different metal centers, are likely to enhance the scope and efficiency of these catalysts. 

Overall, chiral pincer complexes represent a cornerstone of modern asymmetric organometallic catalysis, combining structural precision with functional versatility to enable the efficient and selective construction of chiral molecules. Their ongoing development and application continue to push the boundaries of what is possible in synthetic chemistry, underscoring their importance and potential in both academic research and practical applications.
